# A Sensible Arrangement

**Published:** 1863-09

**Authors:** 


### A SENSIBLE ARRANGEMENT.

THE American Dental Association, and the American Dental Convention held their meetings within a week of each other. And, for some time past, they have held them in the same portion of the country. Many dentists can afford to make one trip across the country, that can not spare the time for two. And many will close their offices, and take a journey when, by so doing they can attend two meetings. Our readers will find it a nice excursion, a profitable journey, to go to Niagara the last week in July, and to Detroit the week following. If the weather proves favorable, an excursion across the lake will be a perfect treat, and we hope a vessel can be chartered for the purpose. But the connection is perfect by rail between the two points, and both are nice places to go to. They are just sufficiently near to tempt everybody (and his wife, of course,) to attend both meetings. The two societies, as we predicted, are blessings to each other. W.

